# A Possible Case of Nitrofurantoin-Associated Immune Thrombocytopenia in a Healthy 45-Year-Old Caucasian Female

**DOI:** 10.7759/cureus.34654

**Published:** 2023-02-05

**Authors:** Caleb Ramey, Alison LePera

**Affiliations:** 1 Internal Medicine, Edward Via College of Osteopathic Medicine, Blacksburg, USA; 2 Emergency Medicine, Edward Via College of Osteopathic Medicine, Blacksburg, USA

**Keywords:** uncomplicated urinary tract infection, antinuclear antibody, idiosyncratic drug reaction, immune thrombocytopenia, thrombocytopenia, drug-induced thrombocytopenia, nitrofurantoin

## Abstract

Patients presenting with immune thrombocytopenia (ITP) may have an associated underlying medical condition or medication exposure serving as the cause of their disease, but oftentimes, ITP is due to an idiopathic, autoimmune cause. While molecular mimicry is recognized as the pathogenesis behind infectious-related causes of ITP, drug-induced ITP is likely due to hapten formation, leading to an inappropriate immune-mediated response. Several drugs are associated with the development of ITP. Nitrofurantoin, a commonly prescribed antibiotic for the treatment of uncomplicated urinary tract infections (UTIs), is a medication not previously associated with the development of ITP, with only one case reporting the development of thrombotic thrombocytopenic purpura (TTP) after nitrofurantoin use. Herein, we report a case of a middle-aged Caucasian female with a history of anxiety and hypothyroidism who developed ITP following exposure to nitrofurantoin three weeks prior to presentation. The patient presented with signs and symptoms consistent with ITP: an isolated low platelet count of 1 x 10^9^/L, petechia, fatigue, normal coagulation parameters, recurrent epistaxis, and melena. Subsequently, she was hospitalized for five days, receiving a total of four units of platelets during her stay. She was started on daily high-dose intravenous corticosteroids and received a one-time dose of intravenous immunoglobulin (IVIG). After achieving a platelet count greater than 30 x 10^9^/L, she was discharged from inpatient care, having responded well to corticosteroid treatment. Upon follow-up with outpatient hematology, her platelet levels were maintained above 150 x 10^9^/L, with full resolution of her acute illness. An autoimmune laboratory workup was negative except for an isolated, newly positive antinuclear antibody IgG with an elevated titer of 1:640, leading to the conclusion that an immunological response to nitrofurantoin had occurred. To our knowledge, this is the first report that describes an association between nitrofurantoin use and ITP. We hope this report aids clinicians in recognizing the various immune-mediated adverse reactions associated with nitrofurantoin.

## Introduction

Immune thrombocytopenia (ITP), also commonly known as idiopathic thrombocytopenic purpura or immune thrombocytopenic purpura, is a type of thrombocytopenia caused by autoantibodies to platelet antigens and is typically an acquired thrombocytopenia [1]. Primary ITP is defined by an autoimmune mechanism leading to platelet destruction and underproduction that is not caused by another medical condition, as is the case for secondary ITP [1]. Moreover, certain medications have been reported as the triggering factor causing ITP, leading to the terminology of drug-induced ITP [2].

ITP is a relatively common hematologic disorder with an incidence highest in the pediatric population [3] with approximately 40% of cases occurring in children younger than 10 years of age [4]. The overall incidence of ITP in the United States is estimated to be approximately 3.3 cases/100,000 persons/year [4]. However, the incidence of drug-induced ITP is rare, with an estimated incidence of 10 per million population; however, information regarding drug-induced ITP may be dependent on the patient population investigated as hospitalized patients, especially those in the intensive care unit, are likely to receive new drugs and may have a higher incidence than healthy outpatients [5].

Antibody production (mainly in the form of IgG) seen in ITP is likely stimulated by CD4 positive T helper cells reacting to glycoproteins on the platelet surface, such as glycoproteins IIb/IIIa [6]. The main antigen-presenting cells are thought to be splenic macrophages with the spleen acting as the primary site of platelet clearance for most patients [7]. In a subset of patients, ITP is thought to occur as a result of an inciting event with several known disease states [8] and medications known to be associated with the development of ITP, with the most common medications being trimethoprim-sulfamethoxazole, quinine, quinidine, rifampin, vancomycin, penicillin, carbamazepine, ibuprofen, ceftriaxone, mirtazapine, oxaliplatin, glycoprotein IIb/IIIa inhibitors, and heparin [9,10]. The mechanism of drug-induced ITP initially involves the drug attaching to the platelet cell membrane; then, the immune system creates antibodies to the target drug-platelet complex (hapten), which results in the removal of the sensitized and opsonized platelet by phagocytes in the spleen [11]. Aside from medications, most secondary causes of ITP can be classified as infectious in nature or due to alterations in immune system function. A type of molecular mimicry is the most likely explanation for ITP development in the setting of viral antigens from pathogens such as human immunodeficiency virus (HIV), hepatitis C [12], and cytomegalovirus [13]. Loss of peripheral tolerance of the immune system in diseases such as systemic lupus erythematosus, chronic lymphocytic leukemia, and immune thyroid disease likely leads to the development of self-reactive antibodies central to the pathogenesis of the disease [14]. Yet, the majority of cases are considered primary or idiopathic with an unrecognized underlying etiology for the disease [8].

The clinical presentation of ITP has extensive variability from case to case, with some patients diagnosed with a chronic, asymptomatic thrombocytopenia and others with symptoms related to thrombocytopenia and bleeding [15]. Mucocutaneous bleeding involving the skin, oral cavity, and gastrointestinal tract is often the most common clinical sign of ITP, with petechia, purpura, and epistaxis commonly reported [15]. ITP, by definition, is a thrombocytopenia with a threshold value of less than 100 x 10^9^/L with the greatest concern for bleeding occurring at platelet counts less than 20 x 10^9^/L [15]. Normal platelet morphology with an enlarged platelet size is commonly noted on peripheral blood smears [15]. Fatigue is another common symptom in those presenting with ITP that may correlate with the degree of thrombocytopenia but not with age, gender, or the duration of ITP [15]. Two distinctive features of ITP are its lack of effect on red and white blood cells and normal coagulation parameters, helping distinguish it from differential diagnoses such as aplastic anemia or TTP [15]. For patients with drug-induced ITP, the typical presentation involves a drop in platelet counts within two weeks of drug exposure [5] and often involves a more abrupt and severe thrombocytopenia with nadir platelet counts often less than 20 x 10^9^/L, with the exception of heparin-induced thrombocytopenia [16].

Even though there are no gold standard tests for the diagnosis of ITP as it is a diagnosis based on the exclusion of other causes of isolated thrombocytopenia [8], a thorough search for any underlying causes of ITP should be completed since the treatment of these conditions can improve platelet counts [8]. This includes the following testing in all patients: complete blood cell count, reticulocyte count, peripheral blood smear, HIV testing, hepatitis B testing, hepatitis C testing, quantitative immunoglobulin level measurement, and blood group (Rh) testing [8]. Bone marrow examination, *Helicobacter pylori* testing, pregnancy testing, thyroid function tests, and antinuclear antibodies (ANAs) may also be obtained in the management of a patient with ITP [8]. For drug-induced ITP, drug-dependent antiplatelet antibodies can be obtained to confirm the etiology, but this test may produce a negative result even in probable drug-induced ITP [16]. The temporal relationship between drug exposure and thrombocytopenia should be assessed, and all possible drug exposures should be reviewed especially in the setting of severe thrombocytopenia [16]. Clinical criteria from the American Society of Hematology indicative of drug-induced ITP include the following: (1) thrombocytopenia was preceded by drug administration with a complete and sustained recovery from thrombocytopenia after drug discontinuation, (2) other medications taken prior to thrombocytopenia were continued or reintroduced after discontinuation of the suspected drug, (3) other causes of thrombocytopenia were excluded, and (4) recurrent thrombocytopenia occurred upon re-exposure to the drug [16].

ITP is generally treated in adult individuals who are not bleeding when platelet counts are less than 20 x 10^9^/L, and, rarely, treatment is required if platelet counts are greater than 50 x 10^9^/L [15]. First-line treatment options for ITP include high-dose corticosteroids, intravenous immunoglobulin (IVIG), and anti-Rh(D) [15]. Second-line therapies may include rituximab, splenectomy, or thrombopoietin receptor agonists, with splenectomy providing the greatest chance for long-term remission [15]. Most patients with ITP will reach a safe and stable platelet count either by spontaneous remission or medication therapy, while others may develop refractory ITP or an associated autoimmune condition [15]. Drug-induced ITP is managed similarly and requires prompt discontinuation of the suspected medication, with platelet counts typically returning to normal within a week of drug cessation [16]. For life-threatening bleeding or severe thrombocytopenia with platelet counts less than 10 x 10^9^/L, platelet transfusions should be promptly administered [8]. Once platelet counts normalize or rise significantly and plateau to a level of at least 50 x 10^9^/L, no additional therapy is needed, and the monitoring time interval can be extended [8].

It is recognized that several drugs may induce the development of ITP [9,10]. Nitrofurantoin, an antibiotic commonly used for the treatment of uncomplicated UTIs, is known to cause hemolytic anemia in patients with glucose-6-phosphate dehydrogenase (G6PD) deficiency and immune-mediated reactions such as autoimmune hepatitis, pulmonary fibrosis, and a lupus-like syndrome. Only one prior case report published in 2012 reported an association between nitrofurantoin use and the development of TTP, a similar but distinct immune-mediated thrombocytopenia [10,17]. To our knowledge, our case report is the only one describing an association between nitrofurantoin use and the development of ITP [10]. Therefore, we present a novel case of possible drug-induced ITP due to prior nitrofurantoin exposure in an otherwise healthy 45-year-old Caucasian female.

## Case presentation

A 45-year-old Caucasian female was admitted to the medicine service after a same-day referral from her primary care provider due to an incidental platelet count finding of 1 x 10^9^/L. Upon presentation to the emergency department, her primary symptoms were lightheadedness and generalized fatigue. The patient reported a week-long history of increased bruising along with a new, spotted rash that had developed over her body. She also reported a history of recurrent epistaxis over the past three weeks along with darkened stools but was otherwise in her usual state of health. The patient denied any recent illnesses or hospitalizations, history of gastric disease, exposures to bloodborne pathogens, or any other symptoms unless stated previously. Approximately three weeks prior, she had completed treatment with nitrofurantoin monohydrate/macrocrystals (Macrobid®) for an uncomplicated UTI. Additionally, the patient had recently restarted her levothyroxine 175 mcg after a brief pause in therapy for one week due to expired refills. The patient denied any other recent medication changes, immunizations, or dietary changes (e.g., tonic water). Her past medical history was significant for hypothyroidism, managed with levothyroxine, and anxiety, managed with escitalopram. The patient had a documented intolerance to hydrocodone with no history of alcohol, tobacco, or illicit drug use. Past surgical history and family history were noncontributory.

Upon physical examination, the patient was in no apparent distress with blood pressure of 150/84 mmHg, heart rate of 87 bpm, temperature of 98.6°F, respiratory rate of 17 breaths per minute, and oxygen saturation on room air at 96%. The only significant physical examination finding was a petechial rash extending over the patient’s arms bilaterally, her torso, and down to her proximal calves. Splenomegaly, lymphadenopathy, or thyromegaly was not present on physical examination. Initial laboratory workup in the emergency department reported her platelet count to be 4 x 10^9^/L, hemoglobin and hematocrit to be within normal limits, a white blood cell count within normal limits, and normal coagulation studies (Table 1). Of note, thyroid stimulation hormone level was 85.62 uIU/mL (reference range: 0.34-5.60 uIU/mL), and her comprehensive metabolic panel was noncontributory. Her blood type was determined to be A positive with a negative antibody screen. Fecal occult blood testing was performed and returned positive. Peripheral blood smear showed rare, scattered, large, immature platelets with no accompanying red blood cell abnormalities. Subsequently, the patient was given one unit of platelets and started on intravenous (IV) dexamethasone 40 mg daily for suspected ITP with a consultation placed to hematology regarding treatment recommendations. A repeat complete blood cell count after the transfusion was administered gave a platelet count of 7 x 10^9^/L and hemoglobin level of 12.7 g/dL (reference range: 12.5-15.2 g/dL). Another transfusion of one unit of platelets was then given to the patient. Her home dose of levothyroxine was also given and continued daily throughout her hospital stay.

**Table 1:** Patient coagulation laboratory results

Coagulation Laboratory Test	Value
Prothrombin time	11.7
Activated partial thromboplastin time	34.1
Prothrombin time/international normalized ratio	1

The following morning, the patient’s clinical status remained unchanged with all examination findings and vital signs stable. Her repeat platelet level on day 2 of being hospitalized resulted in a platelet count of less than 3 x 10^9^/L. A third transfusion of one unit of platelets was then administered to the patient (total of three units transfused within 24 hours) with a subsequent platelet count of less than 3 x 10^9^/L. She received another dose of IV dexamethasone 40 mg and was premedicated with famotidine, acetaminophen, diphenhydramine, and 500 mL of normal saline prior to receiving a one-time dose of 100 g of IVIG due to a persistently low platelet level on the afternoon of day 2. No infusion reactions were reported by the patient. A repeat platelet count after the IVIG infusion was reported to be 8 x 10^9^/L with another unit of platelets transfused on the following morning on day 3 of her stay. Her subjective symptoms of fatigue and lightheadedness had improved by day 3, and her repeat platelet count resulted at 17 x 10^9^/L. Her hemoglobin remained stable, ranging from 10.5 to 12.0 g/dL. She received her third dose of IV dexamethasone 40 mg and continued to be closely monitored. On day 4 of her hospital stay, her platelet count continued to rise to 19 x 10^9^/L, and she continued to be managed with IV dexamethasone 40 mg. On day 5, her platelet count rose to 57 x 10^9^/L, and an ANA IgG resulted as positive with a titer of 1:640. In accordance with the recommendations from the consulting hematologist, the patient was transitioned to oral prednisone 1 mg/kg/day and discharged from the medicine service due to a platelet count greater than 30 x 10^9^/L. She was instructed at discharge to follow-up with outpatient hematology and her primary care provider within the week for continued management.

Five days following discharge, the patient was evaluated in the outpatient hematology clinic, and her platelet count was 150 x 10^9^/L. An ultrasound of the patient’s spleen was obtained, which demonstrated a normal spleen with no free fluid in the left upper quadrant. A prednisone taper was initiated along with oral vitamin B12 due to a borderline low level at 288 pg/mL. The patient then followed up an additional three times within the period of a month with her platelet counts all greater than 150 x 10^9^/L at each visit and no recurrence of her prior symptoms. An autoimmune workup during this time was negative except for an isolated, persistently positive ANA IgG and a titer of 1:320 (Table 2). Her positive ANA, the diagnostic exclusion of several secondary causes of ITP, and the temporal relationship between nitrofurantoin use and the development of ITP led to the conclusion that an immunological response to this medication had occurred. Her prednisone taper was discontinued at the end of this month-long period due to resolution of her possible drug-induced ITP, and the patient was advised to follow up with the outpatient hematology clinic in three months. The patient did not report any changes in health status from the time of hospital discharge to her most recent follow-up appointment.

**Table 2:** Patient autoimmune laboratory results ANA, antinuclear antibody; ENA, extractable nuclear antigen; RNP, ribonucleoprotein; SS, Sjögren syndrome

Autoimmune Laboratory Test	Value
ANA pattern	Homogenous
ANA titer	1:320
ANA Hep2 IgG	Detected
Scleroderma SCL-70	1
Smith (ENA) antibody	9
RNP antibody	9
SSB/La (ENA) antibody	0
SSA 52/Ro (ENA) antibody	9
SSA 60/Ro (ENA) antibody	4
Jo-1 antibody, IgG	0
Anti-DNA, double stranded	3

## Discussion

Our patient with no significant prior medical history presented with a severe form of ITP after receiving treatment for an uncomplicated UTI. While her clinical manifestations of this disease were classic, a few aspects about this case require further discussion. First, the timeline in which this patient presented appears to vary from the typical time course seen in drug-induced ITP. Her exposure occurred three weeks prior to her presentation to the emergency department, and her platelet counts did not spontaneously resolve after finishing her course of treatment, which may represent a protracted, unique course of disease seen with nitrofurantoin exposure. Another key aspect of this case is the lack of further tests to eliminate other known secondary causes of ITP, such as *H. pylori*, HIV, or hepatitis C testing [8]. Though it is unlikely for a monogamous, healthy 45-year-old female to have the conditions commonly associated with ITP based on her history, physical examination, and laboratory tests, a thorough search should have been conducted to exclude these known inciting events as ITP could have been the presenting feature. Lastly, the development of ITP in the setting of a markedly elevated thyroid-stimulating hormone (TSH) level may confound this patient’s presentation of suspected drug-induced ITP. Her elevated TSH may be due to her underlying hypothyroidism and pause in levothyroxine treatment; however, this is unlikely given the extended elimination half-life of levothyroxine. While ITP is associated with both autoimmune hypo- and hyperthyroidism, one retrospective cohort study by Ioachimescu et al. (n = 80) concluded that achieving a euthyroid state did not lead to durable improvement in thrombocytopenia nor was the long-term course of ITP changed by treatment of the thyroid condition [18].

While some aspects of this case point to a nonpharmacological inciting event or even an idiopathic autoimmune cause, the development of a newly positive ANA test and elevated titer should be considered. ANAs are known to be observed in patients with systemic autoimmune disease and can result from exposure to certain medications [19]. While a positive ANA test does not identify a specific autoimmune disease, their presence often indicates an autoimmune or inappropriate immunological process [19]. In our patient with a negative workup for the development of common autoimmune conditions such as systemic lupus erythematous, systemic sclerosis, Sjögren syndrome, and inflammatory myopathies, the presence of a misdirected, nonspecific immunologic response could be the result of exposure to a new medication, such as nitrofurantoin. However, idiopathic causes of an elevated ANA in the setting of ITP cannot be discounted as prior studies have documented positive ANA results after the development of ITP, which may have prognostic value and assist in determining the risk of developing post-ITP autoimmune conditions [20]. Furthermore, in our patient, the use of drug-dependent antiplatelet antibodies would have added benefit in identifying a more definitive relationship between these two variables [16]. Based on clinical criteria from the American Society of Hematology, our case meets the first and second criterion designating this case as one of possible drug-induced ITP [16].

This case provides valuable insight into the multi-factorial nature of immune-mediated diseases and the diagnostic challenge clinicians often face in discovering an underlying etiology for ITP. We recognize that this case might be one of primary ITP with an associated future development of an autoimmune condition. However, given the patient’s history, diagnostic workup, temporal relationship between onset of symptoms and medication exposure, and newly positive ANA test result with an elevated titer, we conclude that her prior use of nitrofurantoin played a role, but of unknown magnitude, in her development of ITP.

## Conclusions

Drug-induced ITP is a rare type of thrombocytopenia that has been documented for several medications. We reported the case of possible drug-induced ITP in an otherwise healthy 45-year-old Caucasian female after treatment with nitrofurantoin for an uncomplicated UTI. Our patient made a full recovery and responded well to corticosteroid and IVIG treatments with a sustained normal platelet count. To our knowledge, this is the first case reporting an association between nitrofurantoin and ITP. Clinicians should be mindful of the various immune-mediated adverse reactions induced by nitrofurantoin, and future research is needed to further elucidate the relationship between certain medications and the development of drug-induced ITP.
